# Genetic Ablation of CXCR2 Protects against Cigarette Smoke-Induced Lung Inflammation and Injury

**DOI:** 10.3389/fphar.2016.00391

**Published:** 2016-10-25

**Authors:** Chad A. Lerner, Wei Lei, Isaac K. Sundar, Irfan Rahman

**Affiliations:** Department of Environmental Medicine, University of Rochester Medical CenterRochester, NY, USA

**Keywords:** cigarette smoke, inflammation, DNA damage, NF-κB, emphysema

## Abstract

Antagonism of CXCR2 receptors, predominately located on neutrophils and critical for their immunomodulatory activity, is an attractive pharmacological therapeutic approach aimed at reducing the potentially damaging effects of heightened neutrophil influx into the lung. The role CXCR2 in lung inflammation in response to cigarette smoke (CS) inhalation using the mutant mouse approach is not known. We hypothesized that genetic ablation of CXCR2 would protect mice against CS-induced inflammation and DNA damage response. We used CXCR2^−/−^ deficient/mutant (knock-out, KO) mice, and assessed the changes in critical lung inflammatory NF-κB-driven chemokines released from the parenchyma of CS-exposed mice. The extent of tissue damage was assessed by the number of DNA damaging γH2AX positive cells. CXCR2 KO mice exhibited protection from heightened levels of neutrophils measured in BALF taken from mice exposed to CS. IL-8 (KC mouse) levels in the BALF from CS-exposed CXCR2 KO were elevated compared to WT. IL-6 levels in BALF were refractory to increase by CS in CXCR2 KO mice. There were no significant changes to MIP-2, MCP-1, or IL-1β. Total levels of NF-κB were maintained at lower levels in CS-exposed CXCR2 KO mice compared to WT mice exposed to CS. Finally, CXCR2 KO mice were protected from lung cells positive for DNA damage response and senescence marker γH2AX. CXCR2 KO mice are protected from heightened inflammatory response mediated by increased neutrophil response as a result of acute 3 day CS exposure. This is also associated with changes in pro-inflammatory chemokines and reduced incursion of γH2AX indicating CXCR2 deficient mice are protected from lung injury. Thus, CXCR2 may be a pharmacological target in setting of inflammation and DNA damage in the pathogenesis of COPD.

## Introduction

Cigarette smoke (CS) is predominately the driving factor in the etiology of chronic obstructive pulmonary disease (COPD). COPD is characterized by destruction of alveolar wall, inflammatory response, and premature lung aging or cellular senescence (Nyunoya et al., 2006; Moriyama et al., 2010; Yao et al., 2012; Ahmad et al., 2015). Pro-inflammatory mediators such as IL-8 (mouse KC) and MIP-2, act as CXCR2 ligands, which are critical for recruitment of peripheral neutrophils. Elevated neutrophil exudate found in bronchoalveolar lavage fluid (BALF) or neutrophils observed amongst the parenchyma in histological sections are a feature of COPD and may contribute to tissue destruction due to lack of inflammatory resolution. The CXCR2 chemokine receptor is differentially expressed on the surface of certain myeloid and lymphoid cell types and plays a major role in peripheral neutrophil mediated inflammation. Chemotactic cytokines that bind to CXC family receptors mediate recruitment of the immune cells expressing them toward the injured or infected tissue (Belperio et al., 2002; Wareing et al., 2007; Nagarkar et al., 2009). The CXCR2^−/−^ deficient/mutant (knock-out, KO) mouse is defective in neutrophil function and exhibits reduced neutrophil lung infiltration following infection, injury, and exposure to ozone (Johnston et al., 2005; Reutershan et al., 2006). Thus, targeting of CXCR2 has been sought after as a potential therapy to quell inflammation in response to lung injury and infection in order to reduce the potential for excessive tissue damage mediated by neutrophil inflammation (Lomas-Neira et al., 2004; Nomellini et al., 2008; Zarbock et al., 2008; Russo et al., 2009; Leaker et al., 2013). However, the role of CXCR2 in mediating neutrophil inflammatory response to CS is not well understood. We hypothesize exposure of lungs to CS lays the ground work for progression toward COPD through activation of CXCR2 and excessive recruitment of neutrophils. To elucidate the role of CXCR2 in neutrophil recruitment and lung damage in response to CS, we utilized CXCR2^−/−^ mice in our experiments. This model allowed us to further assess the CS mediated response of critical lung inflammatory cytokines, the γH2AX DNA damage signal in the CXCR2^−/−^ background, and examine how NF-κB as a master regulator of lung inflammatory cytokines, is affected by CS in the absence of CXCR2 expression *in vivo*.

## Materials and methods

### Ethics statement and scientific rigor/reproducibility

All experiments for animal studies were performed in accordance with the standards established by the United States Animal Welfare Act, as set forth by the National Institutes of Health guidelines. The research protocol for mouse studies was approved by the University Committee on Animal Research Committee of the University of Rochester.

We used a rigorous/robust and unbiased approach throughout the experimental plans (e.g., *in vivo* mouse model) and during data analysis so as to ensure that our data are reproducible along with full and detailed reporting of both methods and raw/analyzed data. All the key biological and/or chemical resources that are used in this study were validated and authenticated (methods and resources) and are of scientific standard from commercial sources. Our results adhere to NIH standards of reproducibility and scientific rigor.

### Animals

Male C57BL/6J (C57) and CXCR2 knockout/deficient (referred to as CXCR2^−/−^ or KO) mice were purchased from the Jackson Laboratory (Bar Harbor, ME). These mice were housed under a 12:12 light-dark (LD) cycle with lights on at 6 a.m. and fed with a regular diet and water *ad libitum* unless otherwise indicated. For CS exposure, mice were kept in a standard 12:12 (LD) cycle with lights on from 6 a.m. to 6 p.m. throughout the experiment. All of the procedures described in this study were approved by the University Committee on Animal Research at the University of Rochester, Rochester, NY.

### CS exposure

Eight to ten weeks old mice were exposed to acute (3 days) CS using Baumgartner-Jaeger CSM2082i cigarette smoking machine (CH Technologies, Westwood, NJ) in the Inhalation Core Facility at the University of Rochester. For acute CS exposure, mice were placed in individual compartments of a wire cage, which was placed inside a closed plastic box connected to the smoke source. The smoke was generated from 3R4F research cigarettes containing 10.9 mg of total particulate matter (TPM), 9.4 mg of tar, and 0.726 mg of nicotine, and carbon monoxide 11.9 mg per cigarette (University of Kentucky, Lexington, KY). Mice received two 1-h exposures per day, 1 h apart, according to the Federal Trade Commission protocol (1 puff/min of 2-s duration and 35 mL volume) for 3 days (acute exposure). Mainstream CS was diluted along with filtered air and directed into the exposure chamber. Monitoring of CS exposure (TPM per cubic meter of air) was done in real time using a MicroDust Pro-aerosol monitor (Casella CEL, Bedford, UK) and verified daily by gravimetric sampling immediately after the exposure was completed. By adjusting the number of cigarettes used to produce smoke and the flow rate of the dilution air, the concentration of smoke was set at a nominal value (~300 mg/m^3^ TPM).

### Tissue harvest and differential cell count in bronchoalveolar lavage (BAL) fluid

Mice were injected intraperitoneally with 100 mg/kg body weight of pentobarbiturate (Abbott laboratories, Abbott Park, IL) and then sacrificed by exsanguination. The lungs were lavaged 3 times with 0.6 ml of saline via a cannula inserted into the trachea. The aliquots were combined, centrifuged, and the BAL inflammatory cell pellet was resuspended in saline. The total cell number was determined with a hemocytometer, and cytospin slides (Thermo Shandon, Pittsburgh, PA) were prepared using 50,000 cells per slide. Differential cell counts (~500 cells/slide) were performed on cytospin-prepared slides stained with Diff-Quik (Dade Behring, Newark, DE).

### Cytokine analysis in bronchoalveolar lavage

The level of proinflammatory mediators, such as the chemokine keratinocyte chemoattractant (KC), macrophage inflammatory protein 2 (MIP-2), interleukin 6 (IL-6), monocyte chemotatic protein (MCP)-1 and, interleukin 1 beta (IL-1β) in BAL fluid were measured by enzyme-linked immunosorbent assay (ELISA) using respective dual-antibody kits (R&D Systems, Minneapolis, MN) according to the manufacturer's instructions. The results were expressed as pg/ml.

### Protein extraction from lung tissues and quantification

One lobe of the lung tissue (~50 mg) was homogenized (Pro 200 homogenizer, at maximum speed, 5th gear for 40 s) in 0.5 mL of ice-cold RIPA buffer containing complete protease inhibitor cocktail (Sigma). The tissue homogenate was then incubated on ice for 45 min to allow total cell lysis. The homogenate was then centrifuged at 13,000 × *g* for 5 min at 4°C to separate the protein fraction from the cell/tissue debris. The supernatant containing protein was aliquoted and stored at −80°C for Western blotting. This fraction was taken for protein analysis by bicinchoninic acid (BCA) colorimetric assay (Thermo Scientific, Rockford, IL) using BSA as a standard.

### Western blot analysis for NF-κB levels in the lungs

Proteins (25 μg) from lung tissue homogenates, were separated on a 7.5% sodium dodecyl sulfate (SDS)-polyacrylamide gel, transferred onto nitrocellulose membranes (Amersham, Arlington Heights, IL), and blocked using 5% bovine serum albumin (BSA) for 1 h at room temperature. The membranes were then probed with NF-κB (sc-109, Santa Cruz, CA), NF-κB-Phospho-serine536 (sc-101752, Santa Cruz, CA), and GAPDH (sc-365062, Santa Cruz, CA) primary antibody (1:1000 dilution in 5% BSA in phosphate-buffered saline [PBS] containing 0.1% Tween 20) at 4°C for overnight. After three 10-min washing steps, the membrane was probed with suitable secondary anti-rabbit, or anti-mouse, or anti-goat antibody (1:10,000 dilution in 5% BSA) linked to horseradish peroxidase for 1 h, and detected using the enhanced chemiluminescence method (Perkin Elmer, Waltham, MA) and images were taken with Bio-Rad ChemiDoc MP, Imaging system. Equal loading of the gel was determined by quantitation of protein as well as by reprobing the same membranes for GAPDH.

### Immunohistochemistry for γH2AX levels in the lungs

Immunostaining was performed on formalin-fixed, paraffin-embedded lung tissue. Paraffin sections (4 μm thick) were deparaffinized and then rehydrated through series of xylene and graded ethanol. Antigen retrieval was performed by heating in citrate buffer (10 mM Citric acid, 0.05% Tween 20, pH 6.0). Primary antibody was incubated overnight at 4°C with rabbit anti-γH2AX antibody (05-636, EMD Millipore, Darmstadt, Germany) Appropriate fluorescently labeled secondary antibodies (FITC-conjugated anti-mouse 2° antibodies) were used to detect the immune complexes before tissues sections were counterstained with 4′,d-diamidino-2-phenylindole (dapi).

### Statistical analysis

Statistical analysis of significance was calculated using one-way analysis of variance (ANOVA) followed by Tukey's *post-hoc* test for multigroup comparisons using the StatView software or GraphPad Prism. Image J software (Version 1.47, National Institutes of Health, Bethesda, MD) was used for quantification of the number of fluorescent punctate nuclei present in γH2AX immunohistochemistry, and densitometry of Western blot analysis. These results are shown as the mean ± SEM. ^*^P < 0.05, ^**^P < 0.01, ^***^P < 0.001 which were considered as statistically significant.

## Results

### Neutrophil influx in CXCR2^−/−^ mouse exposed to acute cigarette smoke

**Figure 1:**
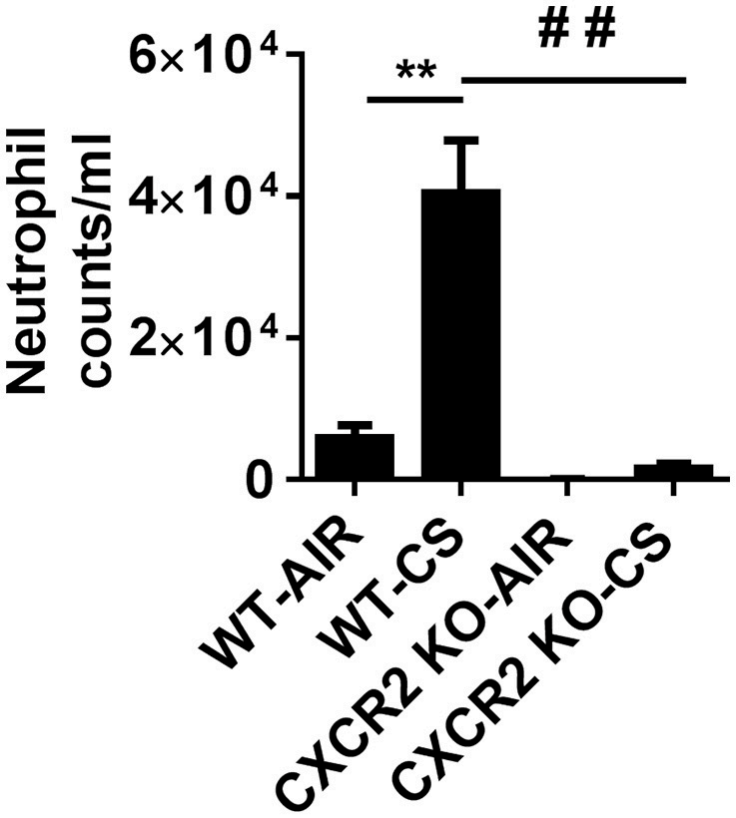
**Elevated neutrophils in BALF via cigarette smoke are blocked in CXCR2^−/−^ mice**. Mice were exposed to acute CS [300 mg/m^3^; total particulate matter (TPM)] for 2 h per day for 3 days. Mice were sacrificed 24 h following last day of CS exposure. Data are shown as the mean ± SEM (WT-Air; *n* = 4, WT-CS; *n* = 5, CXCR2; KO-Air *n* = 3, CXCR2 KO-CS; *n* = 3). ^**^P < 0.01 significant for WT-CS compared to WT-Air; ^##^*P* < 0.01 significant for CXCR2 KO-CS compared to WT-CS.

CXCR2^−/−^ mice exposed with CS for 3 days are refractory to neutrophil influx into the BALF. In contrast, wild type (WT) mice exposed to acute CS exhibit robust neutrophil transmigration into BAL fluid in the lungs compared to WT air group. These results show that CS is a potent activator of neutrophil recruitment to the lung and the process is highly dependent on the expression of CXCR2 (Figure 1).

### Level of pro-inflammatory cytokines in CXCR2^−/−^ mice exposed to acute cigarette smoke

**Figure 2:**
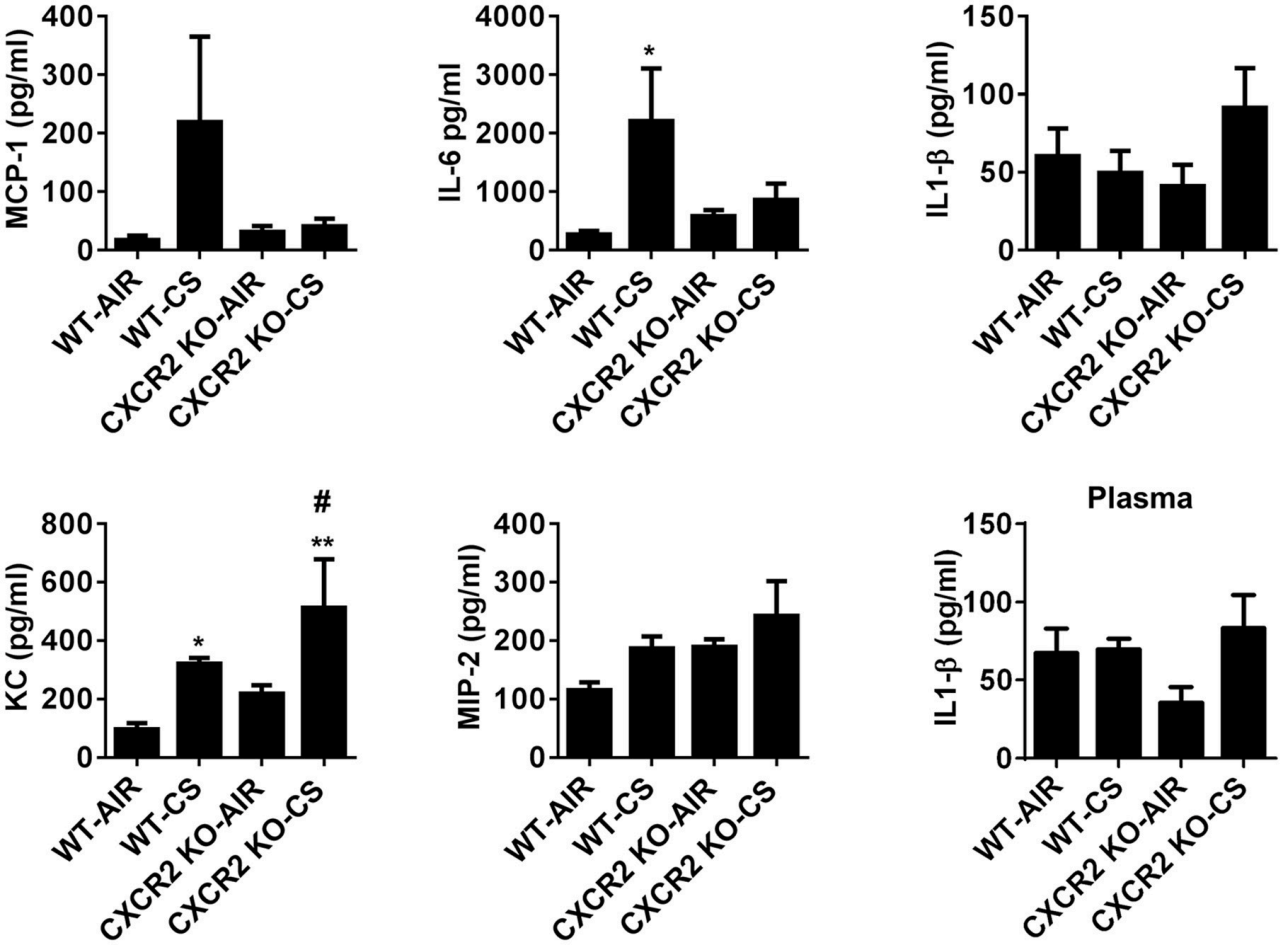
**The levels of proinflammatory mediators in BALF of WT and CXCR2^−/−^ after acute CS exposure**. Mice were exposed to acute CS [300 mg/m^3^; total particulate matter (TPM)] for 2 h per day for 3 days. The levels of proinflammatory mediators in BALF; MCP-1, IL-6, IL-1β, KC, and MIP-2 after 3 days CS exposure and IL-1β in plasma were measured by ELISA. Data are shown as the mean ± SEM (WT-Air; *n* = 3–4, WT-CS; *n* = 5, CXCR2 KO-Air; *n* = 3, CXCR2 KO-CS; *n* = 3). ^*^P < 0.05, ^**^P < 0.01 significant for WT-CS compared to WT-Air; ^#^*P* < 0.05 significant for CXCR2 KO-CS compared to WT-CS.

To assess if the absence of CXCR2 influences levels of pro-inflammatory cytokines that may play a role in neutrophil recruitment in response to acute CS exposure, the Cxcr2 ligands KC and MIP2 were measured in BALF. CXCR2^−/−^ mice exposed to CS exhibit increased levels of KC compared to CXCR2^−/−^ air group and WT air group. Levels of MIP2 are not significantly affected by CS in the WT and CXCR2^−/−^ mice. IL-6 was significantly increased in WT mice exposed to CS. However, in CXCR2^−/−^ mice, IL-6 is resistant to increase by CS. Levels of MCP-1 and IL-1β in CS-exposed CXCR2^−/−^ was not significantly different compared to CS or air exposed WT mice and this was confirmed for IL-1β in plasma (Figure 2). We conclude key cytokines IL-6 and KC both involved in mediating inflammation and neutrophil activity in response to acute CS exposure are altered due to the absence of CXCR2. Lack of neutrophil influx in response to CS results in enhanced KC and blunted IL-6.

### NF-κB expression in CXCR2^−/−^ mouse lung homogenates exposed to acute cigarette smoke

**Figure 3:**
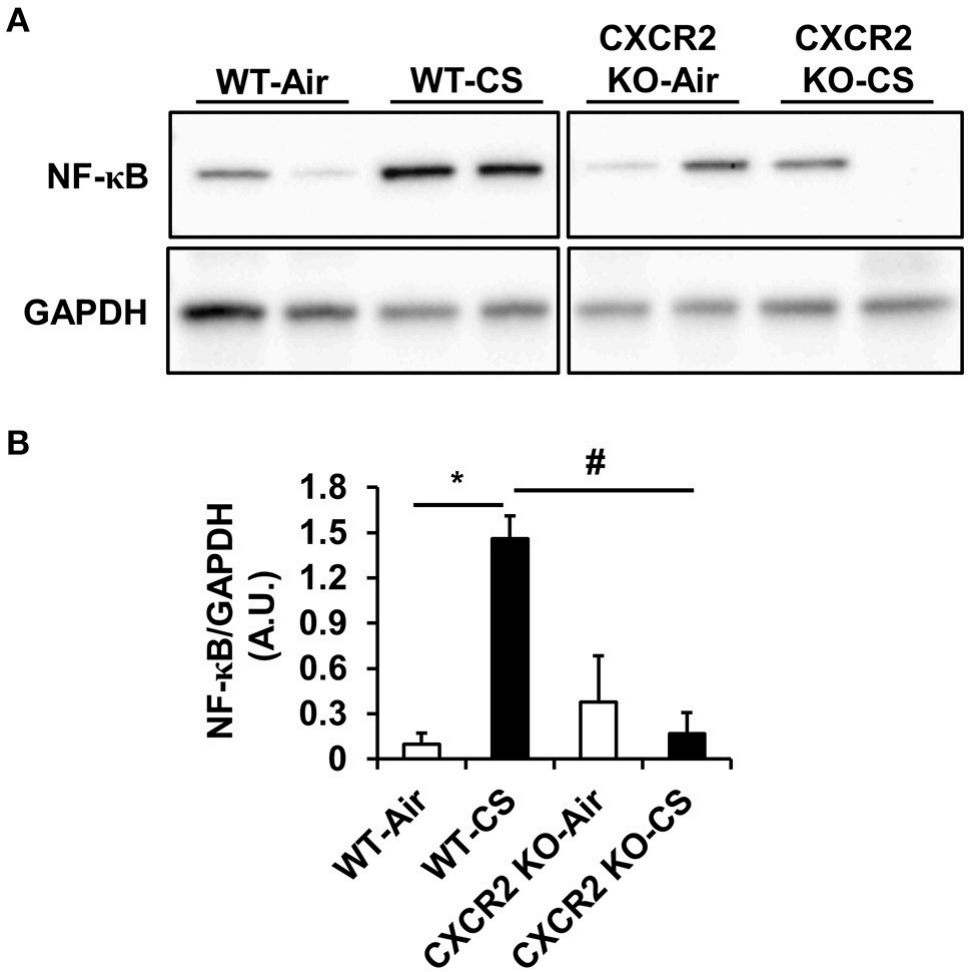
**NF-κB levels in WT and CXCR2^−/−^ mice after acute CS exposure**. Mice were exposed to acute CS [300 mg/m^3^; total particulate matter (TPM)] for 2 h per day for 3 days. Total proteins isolated from lung homogenates were resolved on SDS-PAGE gel for immunoblotting. **(A)** Immunoblot of total NF-κB. GAPDH was used as a housekeeping control. Immunoblots are representative of 2 independent experiments. **(B)** Densitometry for quantitation of relative differences in band intensity for total NF-κB normalized to GAPDH in **(A)**. Measurements are shown as arbitrary units (A.U.). Data are shown as the mean ± SEM. ^*^P < 0.05 significant for WT-CS compared to WT-Air; ^#^*P* < 0.05 significant for CXCR2 KO-CS compared to WT-CS.

To determine if NF-κB is affected in CXCR2^−/−^ mouse lung as it is a master regulator of IL-6, we prepared whole lung homogenates after acute air and CS exposure and measured both the relative levels of total NF-κB and phospho-Ser536 on NF-κB to assess its activation. Following CS exposure in WT mice, total NF-κB levels are increased compared to air group. Conversely, CS is not able to induce lung NF-κB expression to similar levels in CXCR2^−/−^ mice compared to WT CS-exposed mice (Figures 3A,B). Levels of Ser536 phospho-NF-κB relative to total NF-κB remained unchanged between air and CS-exposed WT or CXCR2^−/−^ mice (data not shown). These results suggest that by ablating CXCR2, NF-κB expression in response to CS exposure is attenuated.

### DNA damage signaling in CXCR2^−/−^ mouse lung

**Figure 4:**
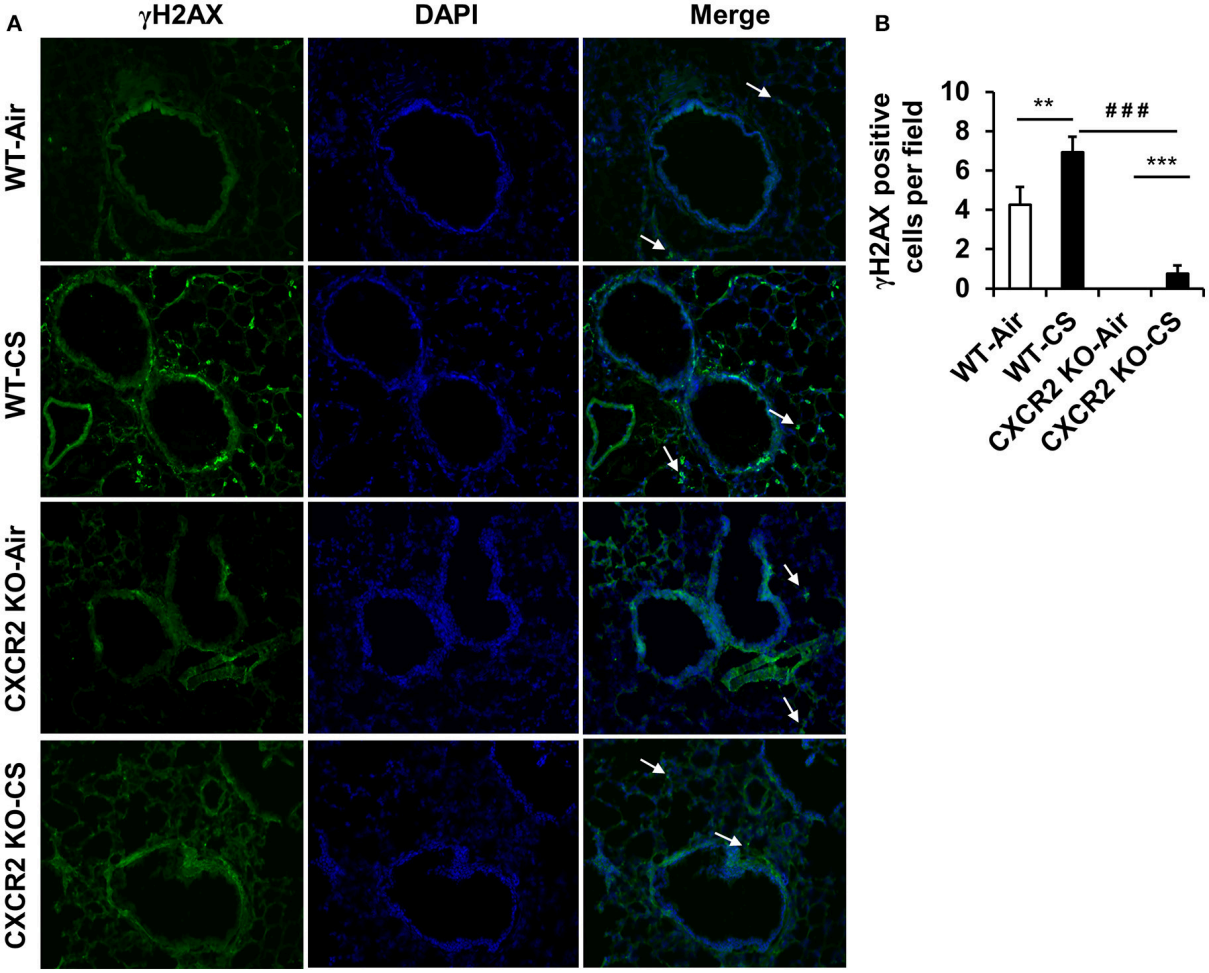
**Assessment of γH2AX positive cells in lung tissues from WT and CXCR2^−/−^ mice after acute CS exposure**. Mice were exposed to acute CS [300 mg/m^3^; total particulate matter (TPM)] for 2 h per day for 3 days. Paraffin embedded lung tissue sections from 24 h following last day of CS exposure were used for immunohistochemistry. **(A)** Representative immunofluorescent images at 20x showing γH2AX positive nuclei (green) overlaid with DAPI stained nuclei (Blue). White arrows point to γH2AX positive regions. **(B)** Quantitation of the number of γH2AX positive nuclei per image for each condition. Data are shown as the mean ± SEM (WT-Air; *n* = 14, WT-CS; *n* = 15, CXCR2 KO-Air; *n* = 10, CXCR2 KO-CS; *n* = 5). ^**^P < 0.01 significant for WT-CS compared to WT-Air; ^***^P < 0.001 significant for CXCR2 KO-CS compared to CXCR2 KO-Air; ^###^*P* < 0.001 significant for CXCR2 KO-CS compared to WT-CS.

Lung tissue is prone to DNA damage by exposure to CS. In addition, since neutrophil influx is suggested to contribute lung DNA damage through inducing genotoxic stress (van Berlo et al., 2010), we sought to determine if CXCR2 status, in its ability to influence CS mediated neutrophil influx into BALF (Figure 1) might affect DNA damage response, which also intersects inflammatory signaling pathways (McCool and Miyamoto, 2012). In lung tissue sections from mice exposed to acute CS, WT mice exhibit an increased number of γH2AX positive cells by immunofluorescence compared to air group. The CXCR2^−/−^ mouse lung was highly devoid of γH2AX positive cells following CS exposure and exhibited only a marginal increase in γH2AX compared to CXCR2^−/−^ air group (Figures 4A,B). These data indicate the γH2AX DNA damage signal is associated with increased BALF neutrophils in response to CS exposure, which are both dependent on expression of CXCR2.

## Discussion

The increased neutrophilic environment induced by CS may contribute to COPD etiology which is typically associated with COPD. COPD is frequently accompanied by bacterial and viral infections that further exacerbate symptoms and accelerate pathogenesis. We utilized CXCR2^−/−^ mice to assess neutrophil recruitment, pro-inflammatory cytokines, NF-κB activity, and the DNA damage signal γH2AX in mouse lungs exposed to acute CS. MIP-2 and KC are primarily CXCR2 ligands. In our CXCR2^−/−^ mice exposed to acute CS, KC levels were significantly altered. The up-regulation of KC in CXCR2^−/−^ mice exposed to CS is consistent with a previous report which showed inhibition of CS-induced lung inflammation by a CXCR2 antagonist (Thatcher et al., 2005), though no gene deletion approach was used without implicating DNA damaging response. CXCR2 is desensitized by very high levels of KC, which may allude to its regulation under inflammatory states where KC is potentially internalized (Wiekowski et al., 2001; Rose et al., 2004). We indeed observed KC increase in CS-exposed WT mice. However, in the CXCR2^−/−^ background, we find KC expression increases further in response to CS which may allude to the possibility that KC levels are regulated by receptor-ligand complex dynamics during heightened tissue inflammation. CXCR2 internalization in addition to canonical G-protein coupled receptor desensitization mechanisms and autocrine feedback responses may further limit excessive immunological activity on cells expressing CXC family receptors, a dynamic that requires further investigation.

Although IL-6 is not a CXCR2 ligand, the CXCR2^−/−^ mice when exposed to either air or CS exhibited reduced IL-6 in the BALF which may extend anti-inflammatory effects beyond limited neutrophil recruitment. An interesting dynamic between IL-6 expression and CXCR2 activity is beginning to emerge in studies involving modulating CXC type receptor activity. The CXCR2 antagonist SCH-N is predominantly a CXCR2 antagonist with partial CXCR1 antagonism in primates and almost exclusively a CXCR2 antagonist in rodent (Chapman et al., 2007). Though SCH-N CXCR2 antagonism did not appreciably affect CS-exposed mouse BALF IL-6 levels (Thatcher et al., 2005), mice treated with a nasal instillation of barn dust in conjunction with the dual CXCR1/CXCR2 antagonist CXCL8 (3-74) K11R/G31P exhibited similar results to CXCR2^−/−^ mice BALF IL-6 levels (Schneberger et al., 2015). Thus, it is possible dual CXCR1/2 antagonism is affecting IL-6 regulation and requires more extensive characterization of how other CXC receptors are affected in the CXCR2^−/−^ mice models. The interesting effect of CXCR2 antagonism by antileukinate on PMN extravasation to the lung under septic challenge leads to reduced IL-6 plasma levels (Lomas-Neira et al., 2004). Thus, further assessment will require optimizing therapeutic approaches for CXC receptor family targeting different inflammatory insults in context with cytokine tissue locale.

IL-β is a potent inflammatory mediator. Stevenson et al. observed a time-dependent increase in IL-1β in BALF in rat model at 24 h post-CS exposure (Stevenson et al., 2005). In our mice, 3 days acute CS exposure did not elicit an appreciable increase in IL-1β in the lavage fluid in WT mice and there were no remarkable changes in the CXCR2^−/−^ mice exposed to air or CS. We cannot conclude for certain IL-β response is unaffected by CS or deficiency in CXCR2, rather the acute exposure we employed in this study, may lead to acclimation or impairment for certain inflammatory targets.

The major role NF-κB plays in modulating inflammation in response to injury and infection in multiple tissues was of interest to us in the CXCR2^−/−^ mice exposed to CS, particularly since we observed differential regulation in KC and IL-6. In NF-κB deficient mice, CXCR2 mediated neutrophil influx into the lung, is reported to be enhanced indicating a regulatory interaction (though potentially indirect) may exist between CXCR2 and NF-κB (von Vietinghoff et al., 2010). Prostate cancer cells cultured under hypoxia, also appear to require NF-κB to upregulate CXCR2 RNA (Maxwell et al., 2007). The CXCR2^−/−^ in our study appears to have a reduced capacity to express total levels of NF-κB, however, the activity of NF-κB indicated by phosphorylation of Serine 536 shows it retains competency in its ability to modulate signaling effects despite reduced total expression levels.

The γH2AX DNA damage signal is frequently associated with senescent positive cell. CXCR2 signaling essentially promotes senescence which is hypothesized to be a factor in the pathogenesis of COPD and lung cancer (Acosta et al., 2008). The γH2AX signal is one facet of DNA damage response (DDR), and is frequently used as a corollary to other senescence assays. The hypothesis that COPD etiology integrates accumulation of senescent cells through aging, smoking, or a combination of both explains that the senescent associated secretory phenotype (SASP) contributes to onset or progression of COPD. CXCR2 has been shown to be critical for senescence as many of the SASP factors are CXCR2 ligands (Acosta et al., 2008). More recently CXCR2 is shown to be involved in DDR mediated senescence (Guo et al., 2013). Our results along with others indicate DDR is engaged within the WT lung parenchyma by CS exposure as indicated by increased γH2AX, increased p53 activity, and changes to CXCR2 dynamics and their ligands (Tiwari et al., 2016). The CXCR2-DDR-senescence connection is intriguing and our results show CXCR2 deficiency may protect from DDR in response to acute CS exposure. However, it is not clear how targeting this axis influences the pathogenesis of COPD (along with overcoming steroid resistance) as it is difficult to target CXCR2 while retaining critical host defense and tissue repair capacity.

Other CXC family members such as CXCR3 have also been shown to attenuate CS mediated lung inflammation in a CXCR3^−/−^ mouse model by reducing CD8^+^ T cell toxicity which further suggests targeting of multiple CXC family members may be an approach to optimize in treatment of inflammatory lung diseases (Nie et al., 2008a,b). In the CXCR2^−/−^ background, neutrophils are further impaired from extravasation from bone marrow into circulation which underscores the severity of the genetic deficit (Eash et al., 2010). In contrast, the pharmacological targeting and new precise genetic methods such as CRISPR/Cas9 models of CXCR2 allows direct oversight into dose, location of administration, protein activity, and timing which will also help to determine how chemokine profiles are affected by CXCR2 antagonism and which are most therapeutic while retaining host immune defenses. Nevertheless, our data show that CXCR2 may be a pharmacological target in setting of inflammation and DNA damage in the pathogenesis of COPD.

## Author contributions

CL, WL, IS, and IR conceived and designed the experiments; WL, IS, and CL performed the experiments; IS and WL analyzed the data; CL, WL, IS, and IR wrote and revised/edited the manuscript.

### Conflict of interest statement

The authors declare that the research was conducted in the absence of any commercial or financial relationships that could be construed as a potential conflict of interest.
